# Asia-Pacific venous thromboembolism consensus in knee and hip arthroplasty and hip fracture surgery: Part 3. Pharmacological venous thromboembolism prophylaxis

**DOI:** 10.1186/s43019-021-00100-8

**Published:** 2021-08-12

**Authors:** Satit Thiengwittayaporn, Nicolaas Budhiparama, Chotetawan Tanavalee, Saran Tantavisut, Rami M. Sorial, Cao Li, Kang-Il Kim, The Asia-Pacific (AP) Region Venous Thromboembolism (VTE) Consensus Group, Aasis Unnanuntana, Alvin Tan, Anthony Pohl, Apisak Angsugomutkul, Apisit Patamarat, Arak Limtrakul, Aree Tanavalee, Azhar Merican, Azlina Abbas, Badrul Shah Badaruddin, Boonchana Pongcharoen, Bui Hong Thien Khanh, Chaithavat Ngarmukos, Charlee Sumettavanich, Chavanont Sumanasrethakul, Chavarin Amarase, Chee-Ken Chan, Cheng-Fong Chen, Chong Bum Chang, Christopher Scott Mow, Chumroonkiet Leelasestaporn, Chun Hoi Yan, Dang-Khoa Tran, David Campbell, David Liu, Edi Mustamsir, Edsel Fernandez Arandia, Eun Kyoo Song, G. Ruslan Nazaruddin Simanjuntak, Hirotsugu Muratsu, Hyonmin Choe, Jamal Azmi Mohammad, Jason Chi Ho Fan, Ji Hoon Bae, Ji-Wan Kim, Jose Antonio San Juan, Jose Fernando C. Syquia, Jun-Ho Kim, Ki Ki Novito, Kriskamol Sithitool, Manoon Sakdinakiattikoon, Masaaki Matsubara, Mel S. Lee, Mohamad Zaim Chilmi, Myint Thaung, Myung Chul Lee, Narathorn Kongsakpaisal, Ngai Nung Lo, Nikom Noree, Nobuhiko Sugano, Paphon Sa-ngasoongsong, Pariwat Taweekitikul, Peter Bernardo, Piti Rattanaprichavej, Piya Pinsornsak, Po-Kuei Wu, Pongsak Yuktanandana, Pruk Chaiyakit, Rahat Jarayabhand, Ross W. Crawford, Ryuji Nagamine, Saradej Khuangsirikul, Seng Jin Yeo, Siwadol Wongsak, Srihatach Ngarmukos, Sukit Saengnipanthkul, Supparurk Suksumran, Surapoj Meknavin, Thakrit Chompoosang, Than Win, Thana Narinsorasak, Thana Turajane, Thanainit Chotanaphuti, Thanarat Reancharoen, Tokifumi Majima, Ukrit Chaweewannakorn, Viroj Kawinwonggowit, Viroj Larbpaiboonpong, Wanshou Guo, Weerachai Kosuwon, Wei Chai, William J. Maloney, Yee Hong Teo, Yixin Zhou, Yunsu Chen, Yutaka Inaba, Yutthana Khanasuk

**Affiliations:** 1 Department of Orthopaedic Surgery, Faculty of Medicine Vajira Hospital, Navamindradhiraj University, Dusit, Bangkok, Thailand; 2 Nicolaas Institute of Constructive Orthopaedics Research and Education Foundation for Arthroplasty and Sports Medicine, Medistra Hospital, Jakarta, Indonesia; 3 Department of Orthopaedic Surgery, Faculty of Medicine, Chulalongkorn University and King Chulalongkorn Memorial Hospital, Thai Red Cross Society, Bangkok, Thailand; 4 Department of Orthopaedic Surgery, King Chulalongkorn Memorial Hospital, Bangkok, Thailand; 5 Department of Orthopaedics, Nepean Hospital, Penrith, New South Wales Australia; 6 Department of Orthopaedics, First Affiliated Hospital of Xinjiang Medical University, Xinjiang, China; 7 Department of Orthopaedic Surgery, Center for Joint Diseases, Kyung Hee University Hospital at Gangdong, 892 Dongnam-ro, Gangdong-gu, Seoul, 134-727 South Korea; 8 Department of Orthopaedic Surgery, School of Medicine, Kyung Hee University, Seoul, South Korea

## Statements of Group 3: Pharmacological Venous Thromboembolism Prophylaxis

### **1. Which pharmacological agents are widely accepted for venous thromboembolism (VTE) prophylaxis in knee and hip arthroplasty and hip fracture surgery?**

#### **Recommendation**

The widely accepted pharmacological agents for VTE prophylaxis include aspirin, unfractionated heparin (UFH), low-molecular-weight heparin (LMWH), adjusted-dose vitamin K antagonists (VKAs), synthetic pentasaccharide factor Xa inhibitor (fondaparinux), oral factor Xa inhibitor, and direct thrombin inhibitor (DTI) anticoagulants.

**Delegate vote:** Agree 100%, Disagree 0%, Abstain 0% (Unanimous Consensus)

#### **Justification**

The American College of Chest Physicians (ACCP) guidelines recommend the use of LMWH, low-dose UFH, VKA, fondaparinux, apixaban, dabigatran, rivaroxaban, or aspirin (all Grade 1B) for patients who undergo total knee arthroplasty (TKA) or total hip arthroplasty (THA) [1]. For patients who undergo hip fracture surgery, the guidelines recommend the use of LMWH, low-dose UFH, VKA, fondaparinux, or aspirin (all Grade 1B) [1]. Although many current guidelines have different recommendations, the variations rely on the risk of VTE, bleeding risk, and patient safety [2]. Overall, the ACCP recommends LMWH as an optimal pharmacological agent for VTE prophylaxis in patients undergoing THA, TKA, or hip fracture surgery [1].

The American Academy of Orthopaedic Surgeons (AAOS) guideline could not make absolute recommendations concerning the most effective prophylaxis agents [3]. As a result, the guideline provides orthopedic surgeons with flexibility regarding the use of different prophylactic regimens. When choosing the medicine, orthopedic surgeons should justify VTEs risk, bleeding risk, and patient safety.

Aspirin is safe because it provides a low risk of bleeding with no requirement for routine blood testing [2]. Several studies supported the use of aspirin for preventing VTE [2, 4]. A meta-analysis of using aspirin as a thromboprophylactic agent in knee and hip arthroplasty found that the overall rate of deep vein thrombosis (DVT) and pulmonary embolism (PE) in both groups after using aspirin was 1.2% and 0.6%, respectively, and the rate of major bleeding was 0.3% [2]. The pooled mortality rate was only 0.2% [2]. A recent systematic review, including 11 relevant studies with various dosing regimens, concluded that aspirin could reduce VTE with a low risk of bleeding complication [4].

LMWH was used instead of warfarin and UFH in clinical practice and VTE studies in knee and hip arthroplasty and hip fracture surgery with a particular efficacy [5]. It can be administered once daily without laboratory monitoring or dose adjustment [6]. Therefore, the use of LMWH is more convenient than UFH, and it has the advantage of less heparin-induced thrombocytopenia [6].

A systematic review and meta-analysis in 2019 suggests that fondaparinux is significantly superior to LMWH in reducing VTE for perioperative arthroplasty surgery [7]. However, the authors of that review advise that clinicians be aware of the higher risk of major bleeding, especially surgical site bleeding, with fondaparinux [7]. A meta-analysis of four randomized controlled trials (RCTs) showed that fondaparinux significantly reduced VTE incidence compared with enoxaparin in major orthopedic surgery [8]. The effect was consistent across all types of surgeries and all subgroups [8]. Major bleeding occurred more significantly frequently in the fondaparinux group [8]. However, the incidence of clinically relevant bleeding (leading to death or reoperation) did not differ between groups [8]. Another RCT showed that the fondaparinux group had a significantly lower incidence of VTE than the enoxaparin group in patients with TKA [9]. Major bleeding occurred more significantly frequently in the fondaparinux group, but there were no significant differences between the two groups in the incidence of bleeding leading to death or reoperation or occurring in a critical organ [9].

Direct factor Xa inhibitors work by binding to the active site of factor Xa, blocking the interaction with its substrate [1]. Examples of oral direct factor Xa inhibitors are rivaroxaban, apixaban, edoxaban, and betrixaban [1]. The ACCP recommends rivaroxaban and apixaban in the same manner as fondaparinux [1]. Rivaroxaban is a Food and Drug Administration (FDA)-approved oral direct factor Xa inhibitor that requires no monitoring [1]. There are numerous studies on rivaroxaban’s effectiveness in preventing DVT [10–13]. In a comparison of extended rivaroxaban with short-term enoxaparin in THA, the results showed that DVT, nonfatal PE, and all-cause mortality up to days 30–42 occurred at 2.0% in the rivaroxaban group, compared with 9.3% in the enoxaparin group [11]. When comparing rivaroxaban with enoxaparin in TKA, the results showed that DVT, nonfatal PE, and all-cause mortality up to day 17 occurred at 6.9% in the rivaroxaban group, compared with 10.1% in the enoxaparin group, with a significant difference [11]. An RCT comparing apixaban with enoxaparin in TKA reported lower rates of clinically relevant bleeding with apixaban [14]. Two RCTs using apixaban showed its superior efficacy compared to enoxaparin [15, 16]. Overall, there were no significant differences in the rates of major bleeding between apixaban and enoxaparin [15, 16].

The direct thrombin inhibitor dabigatran works by binding specifically to the active center of thrombin and inactivating free and fibrin-bound thrombin [17]. This process is reversible, leaving a small amount of free and active thrombin to control hemostasis [17]. In the USA, dabigatran etexilate is an FDA-approved oral direct thrombin inhibitor for the prevention of atrial fibrillation and stroke, but not for VTE prophylaxis after TKA and THA [17]. A study of dabigatran indicated that over 12–15 days of treatment, dabigatran etexilate was not as effective as enoxaparin in preventing total VTE and mortality in patients undergoing TKA [17]. However, an RCT study demonstrated that, over 6–10 days of treatment, dabigatran etexilate was noninferior to enoxaparin for the prevention of VTE in patients undergoing TKA [18]. There was no significant difference in the frequency of major bleeding or the overall rate of adverse events between either dose of dabigatran etexilate and enoxaparin [18]. Similarly, another trial in patients undergoing THA with extended VTE prophylaxis reported the same outcomes [19]. Another trial demonstrated that extended prophylaxis with oral dabigatran etexilate was as effective as subcutaneous enoxaparin in reducing the risk of VTE after THA, and superior to enoxaparin in reducing the risk of major VTE, with similar safety profiles [20].

Warfarin was the first oral anticoagulant widely used in the USA and has been in use since 1954 [4]. It is a VKA that inhibits the synthesis of active vitamin-K-dependent coagulation factors [4]. Therapeutic anticoagulation is reached 24–72 h after the initial dose [21]. Usually, 5 or 10 mg of warfarin is given the night before or the night of surgery, and then dosing is adjusted to maintain an international normalized ratio (INR) of 2.0 [21]. The rate of DVT using low-dose warfarin ranges from 35 to 59% [21]. When comparing warfarin to LMWH, LMWH was more effective in preventing DVT formation but showed no difference to warfarin in preventing symptomatic events, including PE [22, 23]. The risks of warfarin include bleeding and multiple drug interactions [21]. The use of warfarin also requires regular monitoring of the INR [21]. Mismetti et al. [24] conducted a meta-analysis of VKAs. They reported that VKAs were more significantly effective than a placebo or no treatment in reducing DVT and clinical PE [24]. However, there was a significantly higher rate of wound hematoma compared to the placebo [24]. By contrast, VKAs were significantly less effective than LMWH in preventing total DVT and proximal DVT [24]. The differences between VKAs and LMWH in major hemorrhage and wound hematoma were not significant [24].

### 2. Is the protocol for pharmacological VTE prophylaxis similar among knee arthroplasty, hip arthroplasty, and hip fracture surgery?

#### Recommendation

No. Although a general protocol for pharmacological VTE prophylaxis can apply to all patients who undergo knee or hip arthroplasty or hip fracture surgery, different prophylaxis protocols should be considered based on the risk stratification of patients.

Delegate vote: Agree 97.2%, Disagree 1.4%, Abstain 1.4% (Strong Consensus)

#### Justification

Recently, the AAOS and the ACCP developed new evidence-based guidelines for venous thromboembolic prophylaxis after total joint arthroplasty (TJA) [1, 3]. Based on a review of the available literature, the AAOS guideline panel was unable to recommend a specific prophylaxis regimen or duration of prophylaxis following routine TJA [3]. The optimal duration of thromboprophylaxis after TKA remains controversial. It is a common practice to administer prophylaxis using LMWH or UFH until discharge from hospital, usually 7–14 days after surgery [25]. International guidelines recommend extending thromboprophylaxis for up to 35 days following major orthopedic surgery, but the recommendation is weak because of moderate-quality evidence [26]. Extended (4-week) prophylaxis with fondaparinux can produce a 96% reduction in risk of DVT and an 89% reduction in risk of symptomatic VTE events relative to perioperative (1-week) prophylaxis [26]. As the only anticoagulant approved in the USA for thromboprophylaxis in patients with hip fractures, fondaparinux offers more effective prophylaxis against VTE without compromising safety [27].

According to the ACCP, patients with hip fractures are at high risk for DVT and PE [1]. In this patient group, the ACCP recommends using LMWH, low-dose UFH, VKA, fondaparinux, aspirin (all Grade 1B), or an intermittent pneumatic compression device (IPCD) (Grade 1C) for VTE prophylaxis, whereas it does not recommend using apixaban, rivaroxaban, or dabigatran [1]. The duration of pharmacological VTE prophylaxis should continue for 28–35 days postoperatively [1]. Jeong et al. [28] compared the clinical efficacy and side-effect profiles of aspirin, dextran 40, and LMWH in preventing thromboembolic phenomena after hip fracture surgery. Among the three pharmacologic agents, they found a low incidence of thromboembolic phenomena, PE, and fatal PE, with no difference in thromboembolic prophylaxis efficacy [28].

Thus, a personalized protocol for pharmacological VTE prophylaxis should be used based on the surgical plan, underlying condition, and risk of thrombosis in each patient.

### 3. Should pharmacological prophylaxis be administered in all Asian patients who undergo knee and hip arthroplasty and hip fracture surgery?

#### Recommendation

In Asian patients with elevated VTE risk who are undergoing knee and hip arthroplasty or hip fracture surgery, a pharmacological prophylaxis should be administered, except in patients with increased bleeding risk or contraindications for pharmacological prophylaxis.

Delegate vote: Agree 89.0%, Disagree 5.5%, Abstain 5.5% (Strong Consensus)

#### Justification

There are several studies to show that Asian ethnicity is associated with a lower DVT and VTE incidence compared to Caucasians [29–32]. However, in all patients with elevated VTE risk, regardless of ethnicity, the pharmacological prophylaxis is recommended [1, 3, 33, 34].

Although there is no validated risk assessment model applied for Asians, there are no studies to show that Asians have a higher bleeding risk than Caucasians after surgery in the presence of anticoagulants such as UFH, LMWH, Fondaparinux, and the new oral anticoagulants. Liew et al. [34] have proposed using the Caprini risk score for VTE prevention in Asians. A mechanical VTE prophylaxis is suitable in patients with a high risk of bleeding [35].

According to the current guidelines, the type of pharmacological prophylaxis should be considered following risk stratification [3]. A pharmacological prophylaxis is contraindicated in active bleeding and untreated congenital coagulopathies, and only an IPCD is recommended for this group of patients [36]. Contraindications for the IPCD include acute thrombophlebitis, suspected DVT, congestive heart failure, pulmonary edema, and leg ischemia due to peripheral vascular disease [3, 33].

In conclusion, a combination of pharmacological and mechanical VTE prophylaxis is recommended for Asian patients who have elevated VTE risk, based on risk stratification. A mechanical prophylaxis could be considered to use alone in patients who have increased bleeding risk.

### 4. Considering VTE risk and bleeding risk, which method of VTE prophylaxis should apply for Asian patients undergoing knee and hip arthroplasty or hip fracture surgery?

#### 4A. Standard VTE risk without bleeding risk

Recommendation: Mechanical prophylaxis alone or pharmacological prophylaxis combined with mechanical prophylaxis

Delegate vote: Agree 97.2%, Disagree 1.4%, Abstain 1.4% (Strong Consensus)

#### 4B. Elevated VTE risk without bleeding risk

Recommendation: Pharmacological prophylaxis combined with mechanical prophylaxis

Delegate vote: Agree 98.6%, Disagree 0%, Abstain 1.4% (Strong Consensus).

#### 4C. Standard VTE risk with bleeding risk

Recommendation: Mechanical prophylaxis alone

Delegate vote: Agree 97.2%, Disagree 1.4%, Abstain 1.4% (Strong Consensus)

#### 4D. Elevated VTE risk with bleeding risk

Recommendation: Mechanical prophylaxis alone or combined with aspirin

Delegate vote: Agree 86.3%, Disagree 8.2%, Abstain 5.5% (Strong Consensus)

#### Justification

The 2008 AAOS guidelines suggest that mechanical prophylaxis has no bleeding risk [37]. Therefore, mechanical prophylaxis methods should remain in place regardless of VTE risk or bleeding risk [37].

The 2018 European guidelines on perioperative VTE prophylaxis suggest the use of aspirin for VTE prevention after THA, TKA, and hip fracture surgery in patients with an increased bleeding risk (Grade 2C) [38].

According to the 2012 ACCP guidelines, it is recommended to use dual prophylaxis with an antithrombotic agent and an IPCD during the hospital stay in patients undergoing major orthopedic surgery (Grade 2C) [1].

The 2018 National Institute for Health and Care Excellence (NICE) guideline recommended using anti-embolism stockings until discharge, combined with LMWH, as an option in patients undergoing elective TKA and THA [39]. For hip fracture, it is recommended to consider IPCD at the time of admission if pharmacological prophylaxis is contraindicated [39]. This should be continued until the patient no longer has significantly reduced mobility [39].

According to the American Society of Hematology (ASH) 2019 guideline, depending on the risk of VTE and bleeding based on the individual patient and the type of surgical procedure, it is recommended to use combined prophylaxis or mechanical prophylaxis alone [36]. For patients considered at high risk of bleeding, the balance of effects may favor mechanical methods over pharmacological prophylaxis [3]. For patients considered at high risk of VTE, combined prophylaxis is favored over mechanical or pharmacological prophylaxis alone [26].

The Scottish Intercollegiate Guidelines Network (SIGN) guideline suggested that patients with increased risk of bleeding should be given mechanical prophylaxis alone [40]. Pneumatic foot pumps can be considered for prophylaxis as an alternative to IPCD in orthopedic surgery patients [40]. However, the guideline did not recommend combined mechanical with pharmacological prophylaxis [40].

A recent study in Singapore showed a low prevalence of VTE using mechanical prophylaxis alone without chemoprophylaxis in patients who underwent TKA. The prevalence of significant VTE was only 0.82% [29]. With proper patient selection, risk stratification, and stringent perioperative protocols, mechanical prophylaxis alone without routine chemoprophylaxis may be enough in Asians undergoing TKA [29].

A systematic review and meta-analysis of 1399 patients from six RCTs concluded that the addition of IPCD with pharmacological prophylaxis had benefits in DVT prevention in patients undergoing both knee and hip replacement [41]. In TKA, the rate of DVT was significantly reduced from 18.7% with anticoagulation alone to 3.7% with combined modalities [41]. In THA, the rate of DVT was reduced from 9.7% with anticoagulation alone to 0.9% with additional mechanical compression, and the rate of DVT was reduced from 8.7% with mechanical compression alone to 7.2% with additional pharmacological prophylaxis but was not significant [41]. By contrast, the incidence of PE could not be interpreted [41].

In an Asian study, an RCT of Chinese patients, the patients were randomized into four groups to receive graduated compression stockings (GCSs) alone (group A), GCS + LMWH (group B), GCS + IPCD (group C), and GCS + IPCD + LMWH (group D) [42]. The overall incidence of DVT was 5.1% [42]. Group A had the highest incidence of DVT (8.8%), followed by group C (5.2%), group B (3.8%), and group D (2.6%) [42]. There was a significant difference in the incidence of DVT between groups A and D [42]. The incidence of DVT was significantly lower in LMWH-treated patients (group B and group D) than in non-LMWH-treated patients (group A and group C) [42]. The authors concluded that a combined mechanical and pharmacological VTE prophylaxis showed better effectiveness of VTE prevention than mechanical or pharmacological prophylaxis alone [42].

A systematic review of major orthopedic surgeries showed that LMWH with a mechanical device had a significantly decreased risk of total DVT compared to LMWH alone in patients undergoing THA [43]. In patients with TKA, the antiplatelet with mechanical device group had a lower incidence of total DVT than the group in which antiplatelets alone were used [43].

In a case series of 38 patients who underwent lower extremity orthopedic surgery with the highest risk of both venous thrombosis and bleeding, a portable pneumatic compression device was used to prevent VTE [44]. The results showed that the incidence of asymptomatic DVT was 5.3%, and that of symptomatic DVT was 2.6% [44]. No major bleeding or adverse events were observed [44]. Also, a retrospective study reviewed all patients with hemophilia A or B who underwent primary TKA and THA using a mechanical prophylaxis without chemoprophylaxis [45]. They found that there was only one hemophilia B patient with clinically significant VTE, for an incidence of 1.02% [45]. Perez Botero et al. [46] reported similar results in a review of patients with hemophilia A or B who underwent 71 TKAs or THAs. Compression stockings were applied to all patients, of whom 10.5% had IPCDs, and 2.5% had LMWH [46]. Only one patient who received LMWH had asymptomatic DVT [46]. The incidence of symptomatic VTE was 0.5% [46]. The authors concluded that hemophilia patients who had a high risk of bleeding could safely use mechanical prophylaxis of DVT alone without chemoprophylaxis [46].

Bleeding risks, such as those caused by acute liver failure, concurrent use of anticoagulants, lumbar puncture/epidural/spinal anesthesia expected within the next 12 h, lumbar puncture/epidural/spinal anesthesia within the previous 4 h, acute stroke, thrombocytopenia, uncontrolled systolic hypertension, or untreated inherited bleeding disorders, are a contraindication for anticoagulants [3, 33]. Previous major bleeding, severe renal failure, concomitant antiplatelet agent, extensive surgical dissection, and revision surgery are general risk factors for bleeding [3, 33]. In patients with bleeding risk, an IPCD is indicated [3, 33].

### 5. Do the dosage and the duration of perioperative tranexamic acid (TXA) affect the pharmacological VTE prophylaxis in patients undergoing knee and hip arthroplasty and hip fracture surgery?

#### Recommendation

No. TXA administration, at the dosage and the duration administered in knee and hip arthroplasty and hip fracture surgery, is not associated with elevated VTE risk for patients without a known history of VTE.

Delegate vote: Agree 95.9%, Disagree 0%, Abstain 4.1% (Strong Consensus)

#### Justification

There is still no conclusive evidence that TXA increases the risk of VTE [47]. Administration of oral, topical, or intravenous TXA in patients without a known history of VTE does not increase the risk of developing VTE compared to placebo during the perioperative episode of a primary TJA [47]. A meta-analysis investigating the impact of TXA administration on the risk of VTE included 77 high-quality and one moderate-quality RCT [47]. Almost all of the studies excluded patients with a history of a thromboembolic event [47]. In RCTs on knee and hip arthroplasty, the results demonstrated no difference in the VTE rate in the TXA group compared with those in the placebo group [47]. Moreover, the clinical practice guideline of the American Association of Hip and Knee Surgeons (AAHKS) gives a “strong” recommendation that the administration of TXA does not increase risk of VTE [48].

For hip fracture surgery, the meta-analysis of 11 RCTs in 892 patients found that DVT significantly occurred in 16 of 423 patients (3.8%) in the TXA group compared with 8 of 431 patients (1.9%) in the control group. This showed that the risk of DVT was similar in both groups, which means that the use of TXA in hip fracture surgery is safe and does not increase the risk for VTE [49].

### 6. Does pharmacological VTE prophylaxis increase the incidence of wound drainage and/or infection in patients undergoing knee and hip arthroplasty and hip fracture surgery?

#### Recommendation

Yes, pharmacological VTE prophylaxis may increase wound drainage. Prolonged wound drainage was reported as a significant predictor of wound infection after THA. However, there is no evidence that anticoagulants for VTE prophylaxis increase the risk of infection in TKA and hip fracture surgery.

Delegate vote: Agree 98.6%, Disagree 1.4%, Abstain 0% (Strong Consensus)

#### Justification

Pharmacological VTE prophylaxis may increase bleeding, leading to increased wound drainage from a suction catheter, and can also lead to increased subcutaneous wound bleeding and an increase in surgical wound drainage from a surgical wound [50]. Patel et al. [50] conducted a retrospective study of 1211 THA patients and 1226 TKA patients. They found that prophylaxis against VTE with LMWH was associated with a significant increase in wound drainage after THA but not after TKA [50]. Also, there was prolonged wound drainage in the group treated with LMWH compared to the groups treated with Coumadin (warfarin) or aspirin and mechanical compression [50]. On the fifth day after the operation, the LMWH group had significantly more wound drainage than the aspirin group [50]. Moreover, there was a significantly strong positive correlation between the length of hospital stay and the number of days until the surgical wound was dry [50]. The correlation was significantly stronger in the THA group compared to the TKA group [50]. Prolonged wound drainage was a significant predictor of wound infection after THA, and each day of prolonged drainage was associated with a 42% increase in the risk of wound infection [50]. However, prolonged wound drainage did not increase the risk of infection in TKA [50].

Agaba et al. [51] conducted a cohort study of VTE prophylaxis in THA. They found that warfarin was associated with the highest number of postoperative complications at 30 days following surgery [51]. The complications of warfarin consisted of incision and drainage (I&D; odds ratio [OR], 2.04), hematoma (OR, 1.95), transfusion (OR, 2.29), PE (OR, 1.72), DVT (OR, 1.53), prosthetic joint infection (PJI; OR, 1.44), and hemorrhage (OR, 1.92) [51]. The apixaban complications were hematoma (OR, 4.0) and hemorrhage (OR, 3.59) during the 30 days following surgery [51]. There were no statistically significant complications associated with enoxaparin, rivaroxaban, or fondaparinux during the 30-day postoperative period [51].

Runner et al. [52] studied databases from 2014 to 2016 in a total of 22,072 primary TJA cases. The study showed that patients receiving prophylaxis with aspirin or a sequential compression device were significantly associated with having no increased complications [52]. The use of prophylaxis with heparin, enoxaparin, warfarin, rivaroxaban, fondaparinux, and all other prophylactic strategies was significantly associated with a higher likelihood of mild thrombosis (0.9% vs. 0.2%), mild bleeding (1.3% vs. 0.4%), moderate thrombosis (1.2% vs. 0.4%), moderate bleeding (2.7% vs. 2.1%), severe bleeding events (1.2% vs. 0.9%), infection (1.9% vs., 1.3%), and death within 90 days (0.7% vs. 0.3%) [52].

Prolonged wound drainage associated with anticoagulation following THA or TKA has been associated with infection and increased length of hospital stay [50, 52]. Previous studies have investigated the risk of bleeding, prolonged wound drainage, and length of hospital stay among current medications, such as aspirin, LMWH, warfarin, rivaroxaban, and fondaparinux, and have not find significant differences in these complications [50, 52]. However, a retrospective study has shown that, except for aspirin, all other chemoprophylaxis agents increase the risk of bleeding [52].

### 7A. Among all available pharmacological agents for VTE prophylaxis, which is the most appropriate for Asians undergoing elective knee and hip arthroplasty?

#### Recommendation

Aspirin is the most appropriate agent for patients with standard VTE risk. Direct oral anticoagulants (DOACs) or LMWH are the most appropriate agents for patients with elevated VTE risk.

Delegate vote: Agree 82.2%, Disagree 8.2%, Abstain 9.6% (Strong Consensus)

### 7B. Among all available pharmacological agents for VTE prophylaxis, which is the most appropriate for Asians undergoing hip fracture surgery?

#### Recommendation

Inconclusive; there is not enough evidence to support the most appropriate agents.

Delegate vote: Agree 93.2%, Disagree 4.1%, Abstain 2.7% (Strong Consensus)

#### Justification

According to the ACCP guideline released in 2012, for patients undergoing TKA or THA, it is recommended to use one of the following: LMWH, fondaparinux, apixaban, dabigatran, rivaroxaban, UFH, VKA, aspirin (all Grade 1B) or an IPCD (Grade 1C) [1]. It is recommended that patients undergoing hip fracture surgery use one of the following: LMWH, fondaparinux, UFH, VKA, aspirin (all Grade 1B) or an IPCD (Grade 1C) [1]. The guideline also recommended using LMWH in preference to the other agents for patients undergoing TKA and THA and hip fracture surgery (Grade 2B) [1].

The NICE guidelines were released in 2018 and recommended aspirin, LMWH, or rivaroxaban for patients undergoing elective knee replacement [39]. For patients undergoing elective hip replacement, the guidelines recommended LMWH or rivaroxaban [39]. For patients undergoing hip fracture surgery, LMWH or fondaparinux was recommended [39].

The ASH guidelines, released in 2019, recommended using aspirin or anticoagulants for patients undergoing TKA or THA [36]. If the anticoagulants are selected, it is recommended to use DOACs over LMWH [36]. If DOACs are not selected, it is recommended to use LMWH rather than warfarin [36]. For patients undergoing hip fracture repair, one should use LMWH or UFH [36].

Although a study from Singapore showed a low prevalence of VTE in patients undergoing TKA without chemoprophylaxis [29], aspirin could be an option for pharmacological prophylaxis in elective knee and hip arthroplasty, which was recommended by the ACCP, NICE, and ASH guidelines. Several meta-analyses suggested that aspirin is safe and shows good efficacy following TJA and that it demonstrated noninferiority to other anticoagulants [2, 53–56]. The relative risk (RR) of VTE after TKA and THA was 1.12 for aspirin compared with other anticoagulants [56]. Comparable findings were observed for DVT (RR, 1.04) and PE (RR, 1.01) [56]. The risk of adverse events, including major bleeding, wound hematoma, and wound infection, was not statistically significantly different in patients receiving aspirin vs. other anticoagulants [56]. When analyzing TKAs and THAs separately, there was no statistically significant difference in the risk of VTE, DVT, or PE between aspirin and other anticoagulants [56]. Aspirin had a VTE risk not statistically significantly different from that of LMWH or rivaroxaban [56]. Aspirin is an inexpensive, widely available, and well-tolerated agent that does not require routine blood tests and causes fewer adverse events, such as hematoma [56]. Because of the lower prevalence of VTE in Asians, with several studies supporting the use of aspirin, it is reasonable to use aspirin as the pharmacological VTE prophylaxis in those who are considered as having a standard VTE risk.

For Asian patients who have elevated VTE risk and undergo elective knee and hip arthroplasty, some studies favored using aspirin [55]. According to the retrospective study of Tan et al. [55] on 60,467 joint arthroplasties, patients were considered high risk by score > 70 points using the VTE calculator described by Huang et al. [57] The authors concluded that the use of warfarin or LMWH in higher-risk patients did not necessarily result in a reduction in symptomatic VTE [57]. By contrast, aspirin administered to higher-risk patients seemed to be as effective as potent anticoagulants and more effective than warfarin [55]. The study of Huang et al. [57] in 30,270 patients concluded that aspirin is as effective as and safer than warfarin for VTE prophylaxis after TJA, even in patients at higher risk of VTE. However, because of the limited evidence of using aspirin for VTE prophylaxis in TKA and THA, it is reasonable that more potent anticoagulants should be considered rather than aspirin for patients with elevated VTE risk.

In patients with hip fractures, immobility has been reported to increase the risk of VTE [58]. Patients with hip fractures should be classified as elevated-risk patients [58]. Although aspirin was only recommended in hip fracture surgery by the ACCP guideline, other guidelines favored other anticoagulant drugs [1]. LMWH was recommended by the ACCP, NICE, ASH, and SIGN guidelines. The ACCP recommended DOACs in elective knee and hip arthroplasty, but not in hip fracture surgery; however, the NICE, ASH, and SIGN guidelines preferred DOACs over LMWH [1, 36, 39, 40, 59]. As most guidelines and systematic reviews supported using anticoagulants rather than aspirin in patients who undergo hip fracture surgery, it is reasonable that more potent anticoagulants than aspirin should be used for this group of patients.

### 8. Which pharmacological agents for VTE prophylaxis are the most cost-effective for Asian patients undergoing knee and hip arthroplasty?

#### Recommendation

In patients without elevated risk for VTE, aspirin is a more cost-effective prophylactic agent than other agents.

Delegate vote: Agree 90.4%, Disagree 1.4%, Abstain 8.2% (Strong Consensus)

#### Justification

Mostafavi et al. compared the cost and health benefits of anticoagulation using warfarin or aspirin following TJA and demonstrated that the use of aspirin provided a higher quality-adjusted life year (QALY) measure and lower cost than warfarin in all ages for both TKA and THA [60]. Schousboe et al. compared the cost-effectiveness of LMWH or 160-mg aspirin for VTE prophylaxis after TJA and concluded that aspirin is a cost-effective choice for VTE prophylaxis following THA for patients with no history of VTE [5]. The preferred choice following TKA depends on age and is uncertain for those younger than 80 years old [5]. Dawoud et al. [61] compared 15 VTE prophylaxis strategies in elective THA with 12 VTE prophylaxis strategies in elective TKA. They concluded that a strategy consisting of LMWH for ten days, followed by aspirin for 28 days, was the most cost-effective for elective THA [61]. A foot-pump strategy followed closely by aspirin (low dose) was the most cost-effective for elective TKA [61].

Based on these three studies, it seems that aspirin is a cost-effective VTE prophylaxis agent and can be used safely in patients without a history of VTE who are at low risk for the development of VTE following knee or hip arthroplasty. Although these three studies did not investigate Asian patients, the Asia-Pacific VTE consensus experts agreed that a similar recommendation could apply to Asian patients.

### 9. In Asian patients, how long should a pharmacological prophylaxis be given for knee and hip arthroplasty and hip fracture surgery?

#### Recommendation

Although the optimal duration of pharmacological prophylaxis remains inconclusive, the recommended minimum duration should be 10–14 days.

Delegate vote: Agree 83.6%, Disagree 9.6%, Abstain 6.8% (Strong Consensus)

#### Justification

The NICE guideline, released in 2018, recommended pharmacological prophylaxis for 14 days in TKA, 28 days in THA, and one month in hip fracture surgery [39].

The ASH guideline, released in 2019, recommended extended antithrombotic prophylaxis over short-term antithrombotic prophylaxis for patients undergoing major surgery (not specific only to orthopedic surgery) [36]. Extended prophylaxis was considered as beyond 3 weeks, and short-term prophylaxis was considered as up to 2 weeks [36].

The SIGN guideline, released in 2010, recommended extended pharmacological prophylaxis for orthopedic surgery (recommendation Grade A) [40]. However, the optimal duration of extended prophylaxis is unclear [40]. The benefit of post-discharge extended prophylaxis with LMWH is higher in THA than in TKA patients [40].

A systematic review comparing prolonged-duration (21 days) with standard-duration (7–10 days) thromboprophylaxis demonstrated that, in THA, there were fewer symptomatic VTE, PE, nonfatal PE, DVT, asymptomatic DVT, and proximal DVT events with prolonged-duration than with standard-duration prophylaxis [62]. In hip fracture surgery, patients who received prolonged prophylaxis had fewer symptomatic objectively confirmed VTE, DVT, proximal DVT, and distal DVT events than those who received standard-duration prophylaxis [62]. However, in TKA, there were no statistically significant differences in any reported outcomes between prolonged-duration prophylaxis and standard-duration prophylaxis [62].

A prospective study with 197 patients who were undergoing elective TKA and THA and having extended LMWH with mechanical prophylaxis for 4 weeks, compared with a historical group of 795 patients with short-term thromboprophylaxis for only 7–11 days, concluded that extended thromboprophylaxis was more effective than short-term prophylaxis [63].

Parvizi et al. showed that the highest prevalence of symptomatic VTE occurs 1 week after TJA. In detail, 81% occurred within three postoperative days, 89% within 1 postoperative week, and 94% within 2 postoperative weeks [64]. They recommended continuing prophylaxis until the end of these periods [64].

By contrast, two recent studies demonstrated different outcomes [65, 66]. The first study, in 2015, a retrospective review of all primary THAs from the National Joint Registry of Denmark, demonstrated that the 90-day risks of VTE were 1.1% (short), 1.4% (standard), and 1.0% (extended) and the risk of major bleeding was 1.1% (short), 1.0% (standard), and 0.7% (extended) [66]. The study concluded that there was no difference in the risks of symptomatic VTE, VTE/death, or bleeding concerning thromboprophylaxis duration [66]. The second study, in 2019, was a retrospective study of all primary THAs from the Nordic Arthroplasty Register Association (NARA), divided into three groups: short (1–5 days), standard (6–14 days), and extended (≥15 days) duration of thromboprophylaxis [65]. The study demonstrated that the 90-day cumulative incidence of VTE was 1.0% for patients with standard treatment, 1.1% for those with short-term treatment, and 1.0% for those with extended treatment [65]. In conclusion, from these two studies, short-duration prophylaxis had comparable effectiveness to extended-duration prophylaxis in THA [65, 66]. However, these evidences were weak because of the observational study designs from the National Joint Registry.

### 10. What is the optimal dose, starting time, and duration of aspirin usage for VTE prophylaxis in Asian patients who undergo knee & hip arthroplasty?

#### Recommendation

Low dose aspirin ranging from 81-162 mg/day is sufficient for VTE prophylaxis. However, the starting time of administration and duration of usage of aspirin remains inconclusive.

Delegate vote: Agree 97.3%, Disagree 0%, Abstain 2.7% (Strong Consensus)

#### Justification

A recent systematic review categorized low-dose and high dose of aspirin prophylaxis for VTE based on 162mg of aspirin use [67]. In the low dose group, dosages of aspirin ranged from 75 mg daily to 160 mg daily [67]. In the low dose group, two studies looked at Asian patients [68, 69]. These two studies were done in China, using aspirin 100 mg daily [68, 69]. Both studies demonstrated that aspirin 100 mg daily had a comparable effect for VTE prophylaxis as LMWH [68, 69].

There were no significant differences in symptomatic PE, symptomatic DVT, 90-day mortality, or major bleeding between patients receiving low-dose or high-dose aspirin for the entire systematic review [67]. Compared with warfarin, there was also a significantly higher risk of symptomatic DVT with warfarin compared to low-dose aspirin [67]. The studies included in this systematic review reported various durations of aspirin prophylaxis ranging from 14 days to 6 weeks [67]. They found no significant difference between incidences of PE or DVT and the different durations of aspirin treatment examined (<4 weeks, four weeks, and >4 weeks) [67].

Three studies compared low dose aspirin with high dose aspirin directly in the same population [70–72]. Faour et al. investigated the aspirin prophylaxis for VTE in 2018, and 2019 [70, 71]. The first study included patients with TKA and compared between the low dose group (81mg, twice a day) and high dose group (325mg, twice a day [71]. Also, all patients received pneumatic compression stockings, the duration of treatment was 4-6 weeks [71]. Regression model showed no correlation between aspirin dose and VTE incidence or DVT [71]. The incidence of PE was 0.2% in the high-dose aspirin group compared with 0.4% in the low-dose aspirin group without a significant difference [71]. Bleeding and 90-day mortality was similar between the groups [71]. The second retrospective study in THA patients with low-dose aspirin 81 mg twice a day, high-dose aspirin 325 mg twice a day and 4- to 6-week duration of treatment [70]. After accounting for confounders, regression analyses showed no difference between aspirin doses and the 90-day incidence of symptomatic VTE or symptomatic DVT [70]. Bleeding and 90-day mortality was not different between the groups [70]. In conclusion of these two studies, low-dose aspirin was not inferior to high-dose aspirin for the prevention of VTE after TKA and THA [70, 71]. Parvizi et al [72]. in 2017 performed prospective crossover study including 4,651 patients for aspirin prophylaxis of VTE for 4 weeks based on different dosage of aspirin. The low dose group received 81mg of aspirin twice a day and the high dose group received 325mg of aspirin twice a day.. The incidence of VTE of 0.1% in the 81 mg aspirin group was not significantly different from 0.3% in the 325 mg aspirin group [72]. The incidence of gastrointestinal bleeding or ulceration of 0.3% in the 81 mg aspirin group was slightly, but not significantly lower than the 0.4% in the 325 mg aspirin group [72]. The incidence of acute periprosthetic joint infection was 0.2% in the 81 mg aspirin group compared with 0.5% in the 325 mg aspirin group [72]. The 90-day mortality rate was the same in both groups at 0.1% in the 81 mg aspirin group and 0.1% in the 325 mg aspirin group [72].

In conclusion, low dose aspirin ranging from 81-162 mg/d is not inferior to high dose aspirin of 325-650 mg/d for VTE prophylaxis. The duration of aspirin usage has wide variation from 14 days to 6 weeks. However, the evidence available on the optimal time of administration and the optimal duration of aspirin usage for VTE prophylaxis is of limited quality and remains inconclusive.

### 11A. Should the dose and duration of pharmacological VTE prophylaxis in Asian patients with standard VTE risk be adjusted from the recommended dose and duration by the American or European guidelines?

#### Recommendation

There is evidence that a lower dose of the pharmacologic agent is effective, but there is limited evidence for the administration timing. Therefore, a lower dose with delayed pharmacologic administration can apply in Asian patients with standard VTE risk.

Delegate vote: Agree 87.7%, Disagree 4.1%, Abstain 8.2% (Strong Consensus)

### 11B. Should the dose and duration of pharmacological VTE prophylaxis in Asian patients with elevated VTE risk be adjusted from the recommended dose and duration by the American or European guidelines?

#### Recommendation

There is limited evidence that a lower dose and/or delayed administration of the pharmacologic agent is effective in Asian patients with elevated VTE risk. Therefore, the recommended dose and duration should apply in Asian patients with elevated VTE risk.

Delegate vote: Agree 89.0%, Disagree 5.5%, Abstain 5.5% (Strong Consensus)

#### Justification

There is evidence demonstrating that Asian patients have lower DVT prevalence than Caucasians. Bin Abd Razak et al. [29] showed the prevalence of VTE without chemoprophylaxis in Singaporean patients who underwent TKA and mechanical prophylaxis alone. The prevalence of significant VTE is 0.82%, which is significantly lower than that for Caucasians [29]. Fuji et al. [73] found that the mean body weight of Japanese TKA and THA patients who participated in their study was approximately two-thirds that of their Caucasian counterparts. They adjusted their DVT prophylaxis regimen by decreasing the enoxaparin dose to 20 mg bid and confirmed that it is the proper regimen in Japanese patients [73].

Mihara et al. [74] performed a retrospective study of DVT prophylaxis using low-dose aspirin in patients who underwent THA. The patients were divided into a low-risk VTE group and a high-risk VTE group [74]. Low-risk patients received aspirin for 28 days postoperatively [74]. High-risk patients, such as those diagnosed with obesity and/or with a history of VTE, received anticoagulants (enoxaparin or edoxaban) for five days postoperatively, followed by 100 mg/day of aspirin for 28 days [74]. There was a low incidence of symptomatic DVT and PE [74]. There was no postoperative fatal bleeding or bleeding from any organ, such as gastrointestinal or cerebral hemorrhage [74]. This study concluded that the hospital’s risk-stratified protocol using low-dose aspirin or anticoagulants effectively prevented symptomatic VTE [74]. These results were better than those reported from Western countries [74]. There are two studies from China using 100 mg of aspirin daily [67, 68]. Both studies demonstrated that taking 100 mg of aspirin daily had a comparable effect for VTE prophylaxis as LMWH [67, 68].

Therefore, the usual dose and duration in knee or hip arthroplasty may not be necessary in Asians at standard risk for VTE because of their lower rate of VTE and lower body weight compared to Caucasians.

## Data Availability

Not applicable.

## Abbreviations

AAHKS: American Association of Hip and Knee Surgeons
AAOS: American Academy of Orthopaedic Surgeons
ACCP: American College of Chest Physicians
ASH: American Society of Hematology
DOAC: Direct oral anticoagulant
DTI: Direct thrombin inhibitor
DVT: Deep vein thrombosis
FDA: Food and Drug Administration
INR: International normalized ratio
IPCD: Intermittent pneumatic compression device
LMWH: Low-molecular-weight heparin
NICE: National Institute for Health and Care Excellence
PE: Pulmonary embolism
QALY: Quality-adjusted life year
RCT: Randomized controlled trial
THA: Total hip arthroplasty
TJA: Total joint arthroplasty
TKA: Total knee arthroplasty
TXA: Tranexamic acid
UFH: Unfractionated heparin
VKA: Vitamin K antagonist
VTE: Venous thromboembolism
